# Interactions between particulate matter and bacteria during cowshed PM_2.5_-induced respiratory injury initiates GBP2/Caspase-11/NLRP3-mediated intracellular bacterial defense and pyroptosis

**DOI:** 10.3389/fvets.2025.1631913

**Published:** 2025-07-08

**Authors:** Xiaohui Du, Zhenhua Ma, Yize Sun, Yunna Jia, Xiqing Zhang, Cuizhu Zhao, Xiaojun Liang, Xiuzhen Yu, Yunhang Gao

**Affiliations:** ^1^Department of Veterinary Medicine, College of Animal Science and Technology, Jilin Agricultural University, Changchun, China; ^2^Institute of Animal Science, Ningxia Academy of Agriculture and Forestry, Yinchuan, China; ^3^Institute of Agricultural Mechanization, Xinjiang Academy of Agricultural Sciences, Wulumuqi, China

**Keywords:** the animal farm environment, PM_2.5_, *Pasteurella multocida*, respiratory injury, GBP2, NLRP3

## Abstract

**Introduction:**

Fine particulate matter (PM_2.5_) is an important factor in the induction of a variety of respiratory diseases and associated cellular damage. The composition of PM_2.5_ in the animal farm environments is complex, which poses a significant threat to the respiratory health of both workers and livestock, but the causative mechanisms are unclear.

**Methods:**

In order to investigate targeted treatment options, this study focused on the role of microbial components in cowshed PM_2.5_-induced respiratory damage. Utilizing the common pathogenic bacteria (*Pasteurella multocida*) in cowshed PM_2.5_ as a perspective, the intrinsic connection and interaction mechanism between PM_2.5_ particles and bacterial components were explored through *in vivo* and *in vitro* experiments. Bacterial components can interact with PM_2.5_ and are important factors in the respiratory toxicity of PM_2.5_ in farm animal environments by scanning electron microscopy (SEM), Fourier infrared spectroscopy (FTIR) and Zeta potential measurements.

**Results:**

We demonstrate that Bacteria adhered to PM_2.5_ particles and modified the original surface functional groups characteristics, significantly enhanced toxic effects of PM_2.5_ on cells (including oxidative stress levels, release of inflammatory factors, etc.). Furthermore, PM_2.5_ particles significantly enhanced bacterial intracellular invasion, initiated the guanylate-binding protein 2 (GBP2)-mediated intracellular bacterial defense mechanism, further triggered the non-canonical NLRP3 pathway, and ultimately induced a cascade of inflammatory responses and pyroptosis. To explore therapeutic strategies, siRNA silencing of GBP2 and inhibition of NLRP3 were done; GBP2 silencing initially delayed cytotoxicity, but eventually increased the inflammatory response. However, inhibition of NLRP3 expression maintained cell viability and delayed pyroptosis, with potential as an effective solution for treatment of PM_2.5_-induced lung injury in farm-animal environments.

**Conclusion:**

In conclusion, the results of this study demonstrated the interaction between particulate matter and bacteria during cowshed PM_2.5_-induced respiratory injury and clarified the signaling mechanisms among intracellular bacteria, GBP2, NLRP3, and pyroptosis. These findings provide a theoretical basis for developing therapeutic strategies against PM_2.5_-related respiratory diseases in farm-animal environments.

## 1 Introduction

Fine particulate matter (PM_2.5_) is a global pollution in the air that affects human health (1). There is a clear association between PM_2.5_ and lung disease. According to the relevant statistics, the 3.7 million people in the same area, for every 10 μg/m^3^ increase in ambient PM_2.5_ concentration, physician visits for suspected pneumonia and number of respiratory infections increased by 6.32 and 4.72%, respectively (2). In addition to pneumonia, direct and indirect exposure to PM_2.5_ also a contributes to chronic obstructive pulmonary disease (COPD) (3), asthma (4), pulmonary fibrosis (5) and even lung cancer (6). Therefore, elucidating mechanisms of PM_2.5_-induced damage to the respiratory system and digging up effective strategies for treating PM_2.5_-associated respiratory diseases based on this mechanism are crucial.

Composition, microscopic characteristics, and biological toxicity of PM_2.5_ vary among environments. Research has indicated that industrial activities, including vehicle exhaust and factory emissions, contribute to an increase in the presence of heavy metals and organic compounds in atmospheric PM_2.5_. These components have been identified as the primary contributors to respiratory diseases caused by atmospheric PM_2.5_ (7). However, the composition of PM_2.5_ that induces respiratory diseases is also different in some specific environments. In the farm-animal environments, elevated stocking densities, poor ventilation, the equipment operation, and the frequent animal activity result in PM_2.5_ concentrations that are often maintained at high levels (6), whereas animal feed, feces, feathers, and bedding also affect PM_2.5_, resulting in its higher microbial abundance (8). In addition, due to the smaller aerodynamic diameter of PM_2.5_, it may transport disease-causing microorganisms to the end of the bronchi, thereby facilitating the transmission and induction of animal diseases. This is a major threat to staff health and animal health and production (9). At present, most studies focus on microbiological composition of PM_2.5_ in farm-animal environments. Some studies have found that poultry house PM_2.5_ contains pathogenic genera such as *Staphylococcus* and *Corynebacterium*, and harmful fungi such as *Aspergillus* and *Bombyx mori* (10). Bacterial aerosols in hog house also identified pathogenic bacterial genera such as *Streptococcus*, *Fusobacterium*, and *Pseudomonas* (11). In addition, Bacteria such as *Staphylococcus* and *Streptococcus* were also frequently detected in cowshed PM_2.5_ (12). The present studies have focused on the microbial composition of farmed environmental PM_2.5_. However, the biotoxicity profile at higher PM_2.5_ microbial levels in inducing respiratory damage remains to be elucidated. Moreover, in the molecular mechanisms of respiratory damage caused by PM_2.5_ in animal farm environments, the intrinsic link between microbial components and PM_2.5_ particles are largely unknown.

In our previous study of the microbial diversity of ambient cowshed PM_2.5_ samples, *Pasteurella multocida* (*P. multocida*) was detected in all samples (13). *P. multocida* is a conditionally pathogenic bacterium, airborne transmission is an important means of its transmission, and it is a common causative agent of causing upper respiratory tract diseases (14). *P. multocida* has a wide range of hosts and can cause respiratory infections in a variety of domestic and wild animals, and it is the causative agent of bovine haemorrhagic septicaemia (15), porcine pneumonic disease (16), avian cholera (17), and rabbit pasteurellosis (18), as well as bacteraemia, meningitis, etc., in humans (19, 20). However, there are doubts regarding the role of *P. multocida* in biological toxicity of PM_2.5_, as well as interactions between *P. multocida* and PM_2.5_ and the specific mechanism of respiratory injury under the synergy between *P. multocida* and PM_2.5_.

Organisms have complex self-defense mechanisms when faced with microbial invasion and infection. Among them, guanylate-binding proteins (GBPs), a conserved family of interferon-induced GTPases, which play a pivotal role in the immune system’s defense against bacterial, viral and protozoan pathogens that infect the host (21). In our previous transcriptomics study by the research group, GBP2 was aberrantly expressed in alveolar macrophages by cowshed PM_2.5_ stimulation. GBP2, a member of the GBPs family, participate in host defense against intracellular pathogens (22). Studies have shown that GBP2 lyses vesicular membranes harboring pathogens, thereby facilitating host cell recognition and subsequent immune responses, exhibiting potent antimicrobial activity in both *in vivo* and *in vitro* models (23). Recent studies have suggested that GBP2 is closely linked to the activation of the NLRP3 inflammasome (24), but the mechanism is unclear. NLRP3 inflammasome, a member of the NOD-like receptor family, is widely distributed among various types of immune cells (25). As a key component of the innate immune system, NLRP3 can promote the generation of active Caspase-1, which drives maturation and secretion of inflammatory cytokines IL-1β and IL-18 (26). NLRP3 involves regulation and synergy of complex molecular signaling in cellular metabolic state, oxidative stress, pyroptosis and autophagy (27, 28). However, it is unknown whether GBP2 and NLRP3 have roles in cowshed PM_2.5_-induced respiratory damage. In addition, mechanisms of activation of GBP2 and NLRP3 by cowshed PM_2.5_ and mechanisms of the transmission mechanism among the three are also unknown.

Given the many unknowns about the respiratory damage caused by cowshed PM_2.5_ as mentioned above, we hypothesized that microbial components are the primary reason of the observed cytotoxicity. Therefore, this study established an *in vivo* model of respiratory exposure to cowshed PM_2.5_ to investigate the specific mechanisms underlying the respiratory injury inflicted by cowshed PM_2.5_ on model animals (rats). *In vitro*, a model of synergistic infection of alveolar macrophages by *P. multocida* and PM_2.5_ was established, in order to investigate the intrinsic connections and interaction mechanisms between PM_2.5_ and microbial components. Furthermore, studies on the intrinsic association of GBP2 and NLPR3 were conducted, to determine mechanisms of respiratory damage and cellular defense in the presence of cowshed PM_2.5_. These studies will inform treatment options for PM_2.5_-induced respiratory system diseases in farm-animal environments.

## 2 Materials and methods

### 2.1 PM_2.5_ sample preparation

Based on previous studies by our research group (29), PM_2.5_ was collected using a multi-level flow particulate sampler on cattle farms in Changchun, Jilin Province. These PM_2.5_ samples were analyzed for chemical and microbiological constituents (13). The PM_2.5_ standard (ERM-CZ110) was purchased from the JRC Science Hub and its main components are shown (Supplementary Table S1).

### 2.2 Animal model establishment and ethical statements

Experimental animals were selected 6-week-old specific pathogen free (SPF) grade Sprague Dawley (SD) rats (180–220 g) from Liaoning Changsheng Biotechnology Co, with ad libitum access to water and rat chow. After 1 week to acclimatize, exposure was done in an exposure box, as described in the group’s previous studies (30, 31). During the experiment, rats were allocated into a Control group (exposed to clean air) and a PM_2.5_ exposure group (exposure to 4 times the cowshed environment PM_2.5_ concentration), 6 rats per group. The rats of the PM_2.5_ exposure group were exposed to PM_2.5_ for 6 h a day for 30 days, to be able to simulate the daily exposure patterns of animals in the cowshed. At the end of the experiment, to be able to simulate the daily exposure patterns of animals in the cowshed. Rats were anaesthetized by intraperitoneal injection of 3% sodium pentobarbital (40 mg/kg).

An ethical review of animal welfare was conducted by the Animal Experimentation Ethics Committee at Haihua Biotechnology Group Co., Ltd., who adhered to the ARRIVE guidelines and the Chinese National Standard Laboratory Animal Guidelines. The protocol was approved (Animal Experimentation Ethics Number: AUP-20231117-001).

### 2.3 Histopathological examination

Portions of lung were fixed in 4% paraformaldehyde, embedded in paraffin, cut into 5 μm sections, stained with hematoxylin and eosin (H&E), and observed with made a microscope (Phenix, China). Three samples were randomly selected from each group for histopathological assessment.

### 2.4 Lung wet/dry ratio

Immediately after death, a portion of the lung tissue of each mouse was excised and wet and dry, weights (after drying in an oven at 65°C for 24 h) were recorded and used to detect pulmonary edema (based on the W/D ratio of the lungs).

### 2.5 Cell model establishment and grouping

Rat alveolar macrophage cells (NR8383) were purchased from Shanghai Cell Bank and cultured in DMEM medium containing 10% fetal bovine serum and 1% penicillin–streptomycin (C0222). Cells were grown at 37°C in a 5% CO_2_ environment and seeded into 6-well plates (10^6^ cells/well). Cells were cultured to 80% density and then required substances were added.

To determine effects of cowshed PM_2.5_ on NR8383 cells, we chose the concentration of cowshed PM2.5 based on the relevant literature (32, 33). These cells were divided into four groups (Control and low-, medium- and high-concentrations of PM_2.5_ [0, 60, 120, and 240 μg/mL, respectively]), and stimulated for 12 h. To avoid other factors influencing the results, cells were infected with a mixture of *P. multocida* and PM_2.5_ standard to mimic the effects of microorganisms during cowshed PM_2.5_ infection. For this, cells were divided into Control group, PM_2.5_ standard group (120 μg/mL), *P. multocida* group (1 × 10^7^ CFU/mL) and Mixture group (PM_2.5_ standard + *P. multocida*, for intuitive comparison, half doses were used for both the PM_2.5_ standard and *P. multocida*), and stimulated for 12 h. In addition. In addition, referring to the relevant literature (34), an NLRP3 inhibitor (MCC950, HY-12815A, MCE, China) was used to validate the role of NLRP3 in cowshed PM_2.5_-induced cellular damage. Cells were divided into the Control group, MCC950 group, PM_2.5_ group, and PM_2.5_ + MCC950 group, and stimulated for 12 h.

### 2.6 Bacterial strains and culture conditions

*Pasteurella multocida* strains were isolated from the cowshed PM_2.5_ samples. The strain was cultured and identified by streaking onto BHI agar plates and incubating at 37°C. Then, single colonies were picked and inoculated into the BHI liquid medium and incubated at 37°C with shaking. In the experiments, sterile and pyrogen-free BHI liquid medium was used to dilute the bacterial solution.

### 2.7 CCK-8

Cell viability was measured using the CCK-8 kit (C0037). For this, NR8383 cells were added to 96-well plates at a density of 5 × 10^3^ cells per well. After 12 h of stimulation under various conditions, 10 μL of CCK-8 was added to each well and incubated for 1 h. The OD values were read at 450 nm on an enzyme labeler. Cell viability was determined in accordance with the manufacturer’s guidelines.

### 2.8 Oxidative stress indices

Kits were purchased from Nanjing Jiancheng Bioengineering Institute. Reactive oxygen species (ROS), malonic dialdehyde (MDA) and superoxide dismutase (SOD) were determined according to the manufacturer’s instructions.

### 2.9 PM_2.5_ surface characterization

To investigate effects of *P. multocida* on surface properties of PM_2.5_. Samples were divided into PM_2.5_ standard group, *P. multocida* group and PM_2.5_ standard + *P. multocida* group. Samples were fixed with 2% glutaraldehyde, dehydrated with anhydrous ethanol, and subsequently transferred to Shanghai Jiao Tong University for scanning electron microscopy acquisition, Zeta potential determination, and Fourier infrared spectroscopy.

### 2.10 Intracellular bacterial levels

Cells were seeded in 12-well plates at a density of 3 × 10^5^ cells/well using antibiotic-free medium and assigned to four groups: Control, PM_2.5_ standard, *P. multocida*, and PM_2.5_ standard + *P. multocida*. Following 8 h of incubation at 37°C in 5% CO₂, the medium was replaced with DMEM containing 50 μg/mL gentamicin to eliminate extracellular bacteria. After 1 h, cells were lysed with 300 μL of 1% Triton X-100. Lysates were serially diluted 10-fold and plated on BHI agar for CFU enumeration.

### 2.11 siRNA transfection

GBP2 siRNA was transfected into NR8383 cells cultured in 6-well plates for 24 h using Lipofectamine 3000 (L3000015) following the manufacturer’s protocol. Three individual siRNAs targeting GBP2 were used. After 12 h, the transfection medium was replaced with complete medium. Western blotting was performed 48 h post-transfection to validate GBP2 silencing efficiency. For experimental treatments, cells were divided into five groups: Control, NC-siRNA, GBP2-siRNA, PM_2.5_, and PM_2.5_ + GBP2-siRNA.

### 2.12 ELISA

The levels of the inflammatory cytokine IL-1β were measured using *ELISA* kit (ml037361) according to the manufacturer’s instructions.

### 2.13 LDH

The levels of the inflammatory cytokine LDH release were measured LDH cytotoxicity assay kit (C0016) according to the manufacturer’s instructions.

### 2.14 Immunofluorescence

Immunofluorescence staining was performed to assess the expression of GSDMD-N in cells after receiving various stimuli. Cells were fixed with 4% paraformaldehyde for 20 min and permeabilized with 0.3% Triton-X 100 for 10 min. After blocking with BSA for 1 h at room temperature, cells were incubated with GSDMD-N antibody (1:100, ab215203) overnight at 4°C. CY3-coupled secondary antibody (1:100, SA00009-2) was incubated for 1 h at room temperature. Following thorough washing, DAPI was used for nuclear staining, and images were captured under a fluorescence microscope (Olympus).

### 2.15 RT-qPCR

Total RNA was extracted from the cells using the Total RNA Extraction Kit (B511311) and reverse transcribed to cDNA using the PrimeScript™ RT Kit (RR047A). RT-qPCR was performed according to the instructions of the RT-qPCR kit. qPCR reactions were performed using TB Green Mix (RR820A). The target gene expression’s CT value was then contrasted with that of the Control group. GAPDH was employed as a housekeeping control. The 2^−ΔΔCT^ method was used to conduct a relative quantitative analysis. The following primer sequences were used (Table 1).

**Table 1:** qPCR primer sequence.

Gene	Forward primer (5′ → 3′)	Reverse primer (5′ → 3′)
*NLRP3*	GAGCTGGACCTCAGTGACAATGC	AGAACCAATGCGAGATCCTGACAAC
*IL-1β*	AGTGAGGAGAATGACCTGTTC	CGAGATGCTGCTGTGAGATT
*IL-18*	CGAACAGCCAACGAATCCCAGAC	TCACAGATAGGGTCACAGCCAGTC
*Caspase-1*	GCACAAGACTTCTGACAGTACCTTCC	GCTTGGGCACTTCAATGTGTTCATC
*Caspase-11*	TTGGGCTATGATGTGGTGGTGAAAG	TGCTGTCTGATGTTTGGTGCTCTG
*GAPDH*	CCTGCACCACCAACTGCTTA	CATCACGCCACAGCTTTCCA

### 2.16 Western blotting

RIPA lysate containing 1% PMSF (C500005) was used to lyse cultured cells to extract total protein. Sample protein concentrations were determined using a BCA protein quantification kit (P0010S), denatured and subjected to SDS-PAGE gel electrophoresis. Proteins were transferred to PVDF membranes (GVWP04700) and blocked with 5% skimmed milk powder for 2 h. Then, the membrane was incubated overnight with the corresponding primary antibody, and secondary antibodies were incubated for 2 h at room temperature. After sufficient washing using TBST solution, an ECL luminescence assay (PW30601S) was performed to assess protein expression. Protein bands were detected using an Amersham Imager 680. Image analysis was performed using ImageJ (Version: 1.54f). The details of the antibodies used are provided in Supplementary Table S2.

### 2.17 Statistical analysis

Each experiment was repeated at least three times and values expressed as the mean ± SD of the measurements, with plots using GraphPad Prism version 8.0 software (GraphPad Software, San Diego, CA, USA). Statistical comparisons were made using unpaired Student’s *t*-tests. A one-way analysis of variance (ANOVA) was used to compare data between multiple groups, and the Tukey–Kramer post-test was used to locate differences. Statistical significance was defined as *p* < 0.05.

## 3 Results

### 3.1 Cowshed PM_2.5_ induced rat lung and *in vitro* alveolar macrophage damage

**Figure 1:**
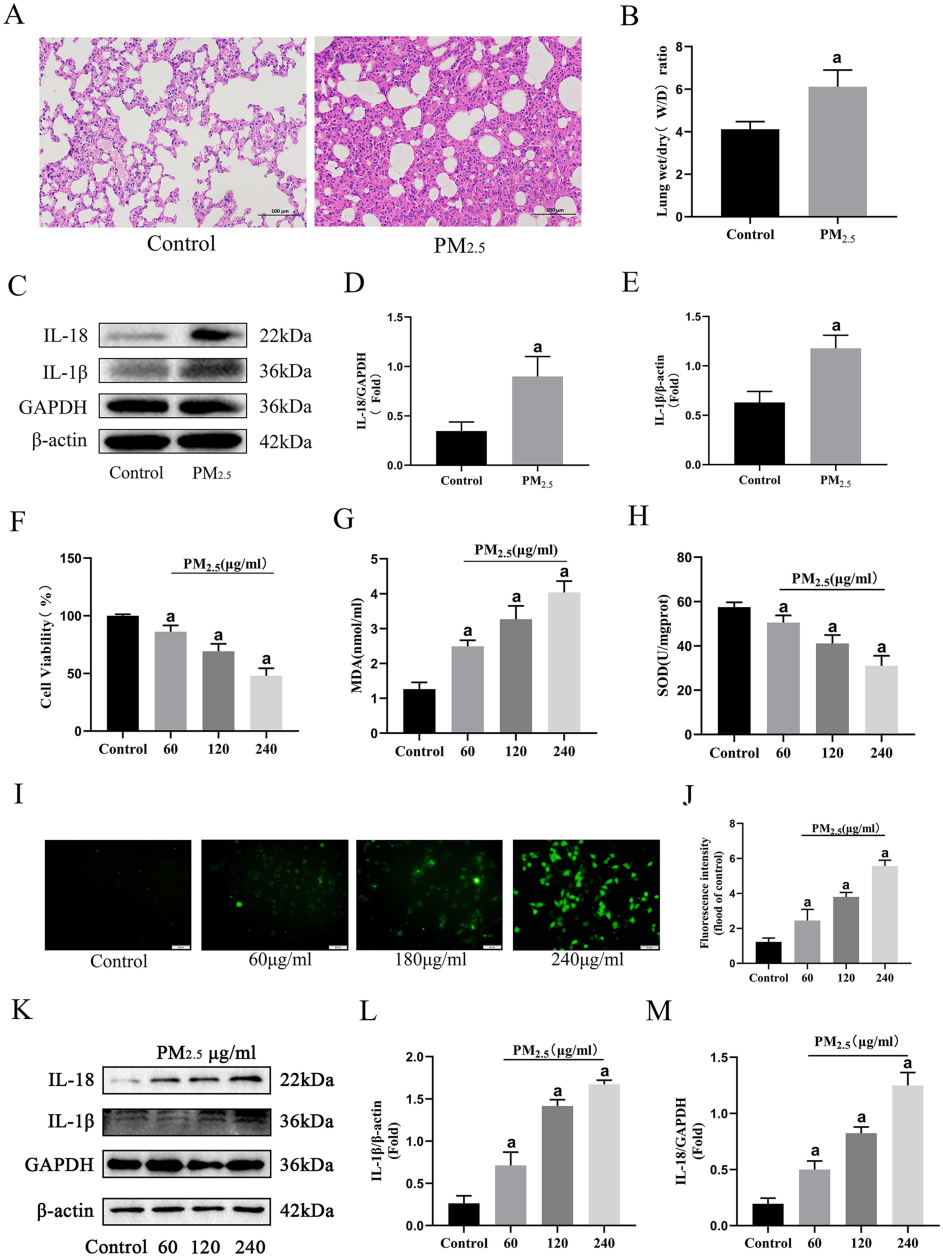
Cowshed PM_2.5_ induced rat lung and *in vitro* alveolar macrophage damage. **(A)** H&E stained sections of rat lungs after cowshed PM_2.5_ exposure treatment. Scale bar = 100 μm. **(B)** W/D ratio in rat lung. **(C–E)** Expression levels of inflammatory factors in rat lungs. **(F)** Cell viability levels of NR8383 cells upon exposure to cowshed PM_2.5_. **(G–J)** Oxidative stress in NR8383 cells during exposure to cowshed PM_2.5_. **(K–M)** Inflammatory factor levels in NR8383 cells during exposure to cowshed PM_2.5_. Results are expressed as mean ± SD deviation of three determinations. ^a^*p* < 0.05, compared to the Control group.

To assess the effects of cowshed PM_2.5_ on the respiratory system, changes of *in vivo* rat pulmonary assessments and *in vitro* alveolar macrophages were independently evaluated after exposure to cowshed PM_2.5_. *In vivo*, in comparison with the Control group, lung tissue obvious pathological damage, including alveolar hemorrhage, alveolar wall thickening, inflammatory cell infiltration, and alveolar interstitial exudates (Figure 1A). Moreover, comparison to the Control group, exposure of rats to cowshed PM_2.5_ significantly increased the lung W/D ratio and concentrations IL-1β and IL-18 (Figures 1B–E).

Given the crucial role of alveolar macrophages in defending against PM_2.5_ pulmonary invasion (35), rat alveolar macrophages (NR8383) was selected for *in vitro* experiments. There was a notable decline in cell viability with as PM_2.5_ concentration increased (Figure 1F). Moreover, cell viability approached 50% at the exposure concentration of 240 μg/mL. Thus, 120 μg/mL was chosen for subsequent experiments. Regarding oxidative stress, there was a significant decrease in SOD and significant increases in MDA and ROS compared to the Control group, (Figures 1G–J). Furthermore, consistent with *in vivo* findings, exposure to cowshed PM_2.5_ significantly elevated IL-1β and IL-18 (Figures 1K–M). In conclusion, the *in vivo* and *in vitro* fundamental data demonstrate that cowshed PM_2.5_ is respiratory toxic and poses a significant threat to animal respiratory health.

### 3.2 Microbiological components are important reason for cowshed PM_2.5_-induced respiratory damage, and bacteria strongly amplified toxic effects of PM_2.5_ particles on cells

**Figure 2:**
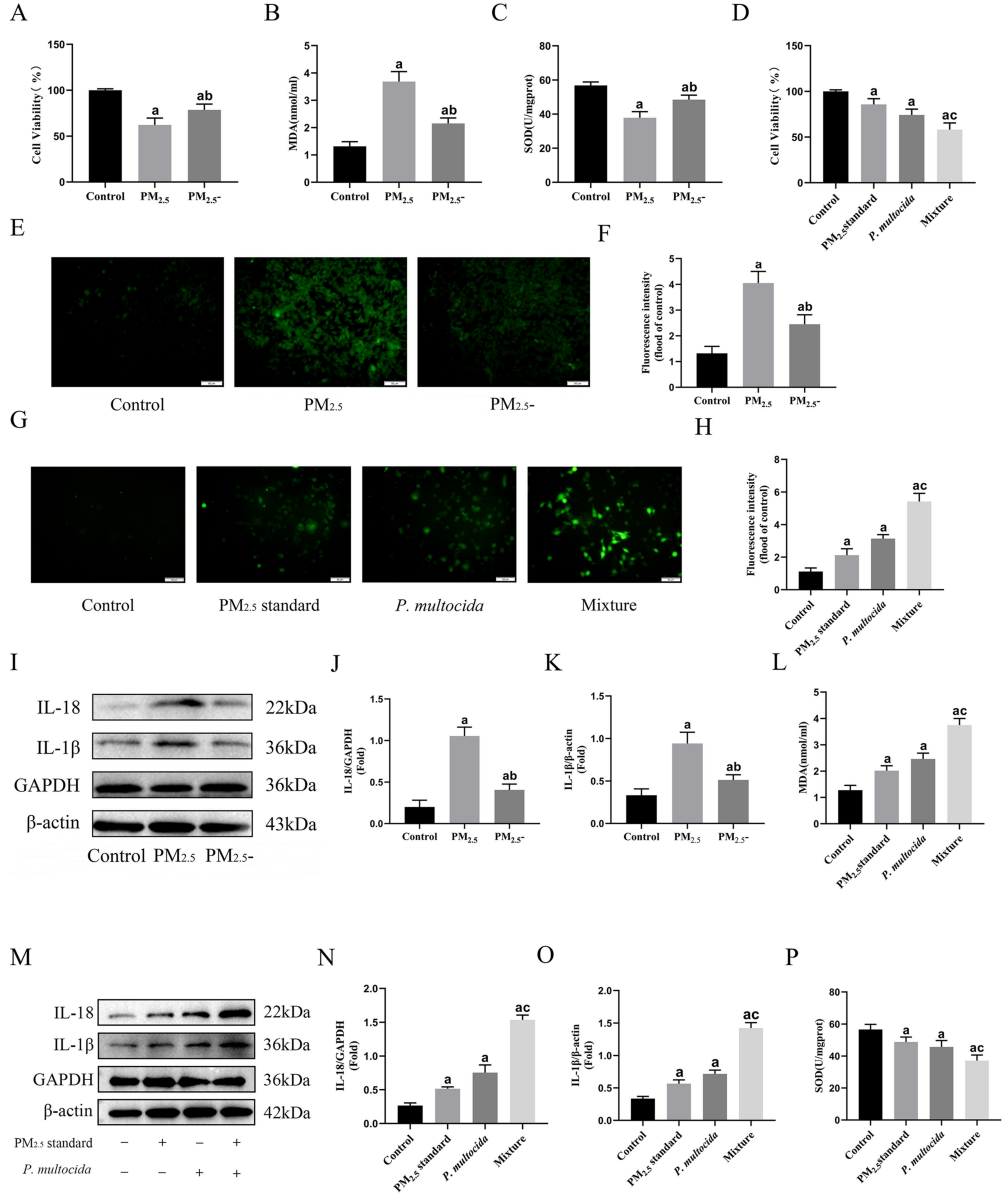
Microbiological components are important reason for cowshed PM_2.5_-induced respiratory damage, and bacteria strongly amplified toxic effects of PM_2.5_ particles on cells. **(A)** Viability of NR8383 cells after inactivation of cowshed PM_2.5_. **(B,C,E,F)** Oxidative stress on NR8383 cells after inactivation of cowshed PM_2.5_. **(D)** Cell viability under the synergistic effect of PM_2.5_ standard and *P. multocida*. **(G,H,L,P)** Oxidative stress in NR8383 in response to the synergistic effect of PM_2.5_ standard and *P. multocida*. **(I–K)** Inflammatory factors in NR8383 cells after inactivation of cowshed PM_2.5_. **(M–O)** Inflammatory factors in NR8383 cells under the synergistic effects of PM_2.5_ standard and *P. multocida*. Results are expressed as mean ± SD deviation of three determinations. ^a^*p* < 0.05, compared to the Control group. ^b^*p* < 0.05, the PM_2.5_-group compared to the PM_2.5_ group, ^c^*p* < 0.05, the Mixture group compared to the PM_2.5_ standard group.

To determine the role of microbial components while minimizing alterations to cowshed PM_2.5_ composition, heat inactivation treatment was done and cellular toxicity assessed. Cell viability exhibited partial restoration (Figure 2A), concomitant with the restoration of ROS, MDA, and SOD contents in the cowshed PM_2.5_ inactivation group (Figures 2B,C,E,F), in comparison to the cowshed PM_2.5_ group. Furthermore, restoration of inflammatory factors IL-1β and IL-18 concentrations was most prominent (Figures 2I–K). The results provide strong evidence that the presence of microorganisms is a significant cause of cowshed PM_2.5_-induced respiratory damage.

The microbial species in cowshed PM_2.5_ are diverse, in order to explore the mechanisms and intrinsic links between microbial components in their induction of cytotoxicity, it is first necessary to clear components and control variables. In this regard, this study used PM_2.5_ standard with well-defined compositions, as well as *P. multocida*, a common pathogenic bacteria in cowshed environments to simulate cowshed PM_2.5_ and reevaluated cellular toxicity. The results showed that although both the PM_2.5_ standard group and the *P. multocida* group showed some cytotoxicity. However, the Mixture group at half the dose showed more significant cytotoxicity. Compared to the PM_2.5_ standard group, the Mixture group had significant decreases in cell viability and the antioxidant factor SOD (Figures 2D,P), significant increases in the oxidant factors MDA and ROS, and a more pronounced increase in the inflammatory factors IL-1β and IL-18 (Figures 2G–H,L-O). These results indicate that the bacterial component appears to strongly amplify the cytotoxic effect of PM_2.5_ during PM_2.5_ stimulation of cells, although at a much lower dose.

### 3.3 Interactions between bacteria and PM_2.5_ particles enhanced intracellular invasion and surface group modification

**Figure 3:**
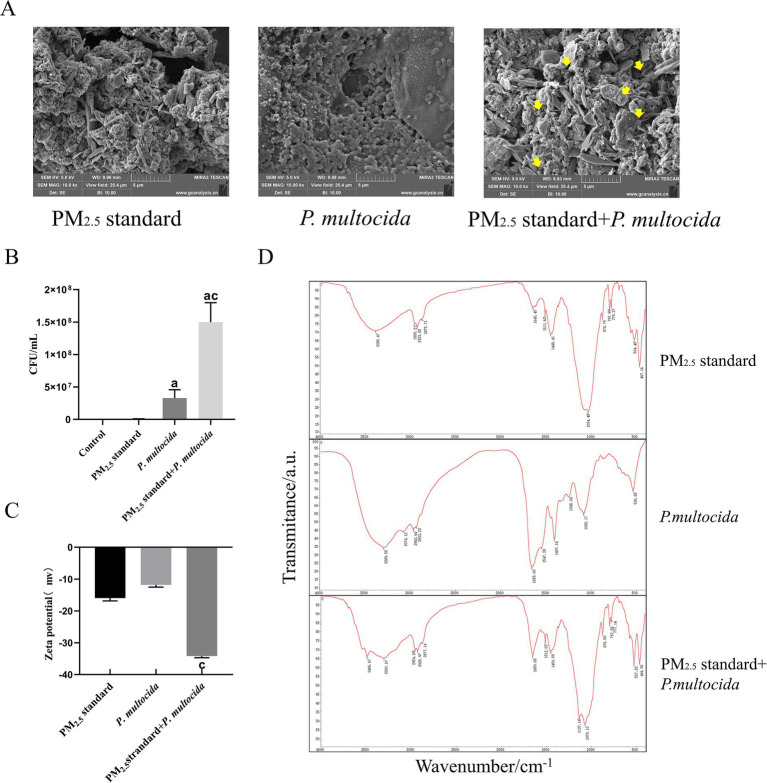
Interactions between bacteria and PM_2.5_ particles enhanced intracellular invasion and surface group modifications. **(A)** Scanning electron microscopy revealed *P. multocida* adhered to PM_2.5_ particles. **(B)** Significant increase in the number of intracellular bacteria after synergistic interaction of PM_2.5_ standard and *P. multocida*. **(C)** Zeta potential shifted significantly in the negative direction after synergistic interaction of PM_2.5_ standard and *P. multocida*. **(D)** Changes in PM_2.5_ surface groups after synergistic interaction of PM_2.5_ standard and *P. multocida*. Results are expressed as mean ± SD deviation of three determinations. ^a^*p* < 0.05, compared to the Control group. ^b^*p* < 0.05, compared to the PM_2.5_ standard group.

To clarify the mechanism of cowshed PM_2.5_-induced respiratory damage, this study focused on the interaction between PM_2.5_ particles and bacteria. Firstly, with regard to effects of PM_2.5_ on bacteria, compared to the *P. multocida* single group, the PM_2.5_ standard + *P. multocida* group showed a significant increase in intracellular bacterial count (Figure 3A), indicating that PM_2.5_ enhances bacterial entry into the cytosol. The elevated viable bacteria in the cytosol may be a key factor for enhanced toxicity. Scanning electron microscopy further revealed that PM_2.5_ particles could adhere to and encapsulate bacteria (Figure 3B), directly explaining the increased intracellular bacterial load in the PM_2.5_ standard + *P. multocida* group.

In addition, whether the bacterial component also alters the surface properties of PM_2.5_ particles is unclear. To evaluate bacterial effects on PM_2.5_ particles, we first measured Zeta potential changes. The findings indicated a notable negative shift in zeta potential in the Mixture group in comparison with the PM_2.5_ standard group (Figure 3C). The significant changes in zeta potential indicate greater stability within the system (36). This suggests that the bacterium enhances the anti-aggregation and anti-deposition properties of PM_2.5_ particles. Subsequently, every group was subjected to analysis by FTIR. The results demonstrated that the absorption peaks of the PM_2.5_ standard included O–H and methyl stretching vibrations, etc., whereas absorption peaks of *P. multocida* included antisymmetric and symmetric stretching vibrations of amino N–H, and stretching vibrations of amide C=O, amongst others. In comparison to the PM_2.5_ standard group, the absorption peak at 3,385 cm^−1^ in the PM_2.5_ standard + *P. multocida* group was red-shifted towards 3,300 cm^−1^. The change there suggests that bacterial cells induced elongation of O–H and N–H bonds in the PM_2.5_ standard + *P. multocida* group, leading to hydrogen bond association. The enhanced absorption peak at 1,650 cm^−1^ and the emergence of a new peak at 527 cm^−1^ suggest specific binding between bacterial organic groups and PM_2.5_ particles, which potentiates the biological toxicity of PM_2.5_ (Figure 3D). These alterations in the peaks of absorption indicated that *P. multocida* modified the surface groups of the PM_2.5_ standard. The results supported the hypothesis that interactions occurred between the bacterium and PM_2.5_ particles and indicated that the two were mutually promoting in terms of enhancing cytotoxicity.

### 3.4 Cowshed PM_2.5_ activates cellular GBP2 expression in response to defense against intracellular bacteria

**Figure 4:**
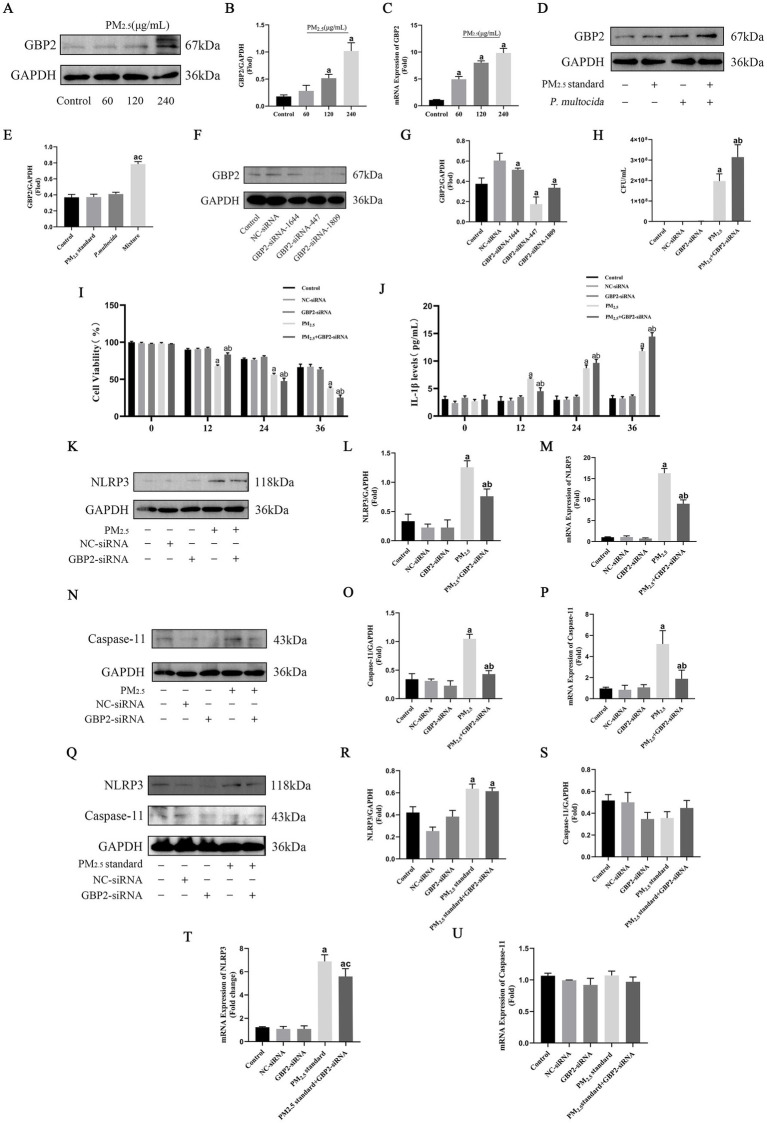
Cowshed PM_2.5_ activates cellular GBP2 expression in response to defenses against intracellular bacteria, whereas recognition of intracellular bacteria by GBP2 activates Caspase-11-mediated non-classical NLRP3. **(A,B)** Expression of GBP2 protein in NR8383 after cowshed PM_2.5_ treatment was detected by Western blotting. **(C)** Expression of GBP2 mRNA in NR8383 after cowshed PM_2.5_ treatment was detected by qRT-RCR. **(D,E)** Expression of GBP2 under the synergistic effect of PM_2.5_ standard and *P. multocida*. **(F,G)** Silencing effect was verified for three siRNAs of GBP2. **(H)** The number of intracellular bacteria after interference with GBP2 under the influence of cowshed PM_2.5_. **(I)** Changes in cell viability over time after interfering with GBP2 under the influence of cowshed PM_2.5_. **(J)** Changes in IL-1β over time after interfering with GBP2 under the influence of cowshed PM_2.5_. **(K–M)** Decreased expression of NLRP3 after interference with GBP2 under the influence of cowshed PM_2.5_. **(N–P)** Decreased expression of Caspase-11 after interference with GBP2 under the influence of cowshed PM_2.5_. **(Q–U)** Interference with NLRP3 and Caspase-11 expression after interference GBP2 under the influence of PM_2.5_ standard. Results are expressed as mean ± SD deviation of three determinations. ^a^*p* < 0.05, compared to the Control group. ^b^*p* < 0.05, compared to the PM_2.5_ group, ^c^*p* < 0.05, compared to PM_2.5_ standard group.

Based on the finding that PM_2.5_ increases the probability of bacteria entering the cytosol as an important cause of cytotoxicity. This study focuses on digging into the signaling of alveolar macrophages to intracellular bacterial defenses to investigate the specific mechanisms of cowshed PM_2.5_ respiratory toxicity. Based on a bioinformatics screen for cowshed PM_2.5_-induced cellular differential genes, there was a significant increase in guanylate binding protein 2 (GBP2) expression in the lung under the role of cowshed PM_2.5_. Under the role of cowshed PM_2.5_, the mRNA and protein expression levels of GBP2 are shown (Figures 4A–C). However, whether the high expression of GBP2 was induced by an increase in intracellular bacteria and whether it was involved in the host cell defense process against gram-negative cytoplasmic bacteria was not clear. Firstly, still used PM_2.5_ standard and *P. multocida* to simulate cowshed PM2.5. In comparison to the Control group, neither PM_2.5_ standard nor *P. multocida* groups effectively activated GBP2 expression. However, GBP2 expression was significantly higher in the Mixture group (Figures 4D,E). This result provides substantial evidence that, under the influence of PM_2.5_ in the cowshed, GBP2 expression is induced by an increase in intracellular bacteria.

**Figure 5:**
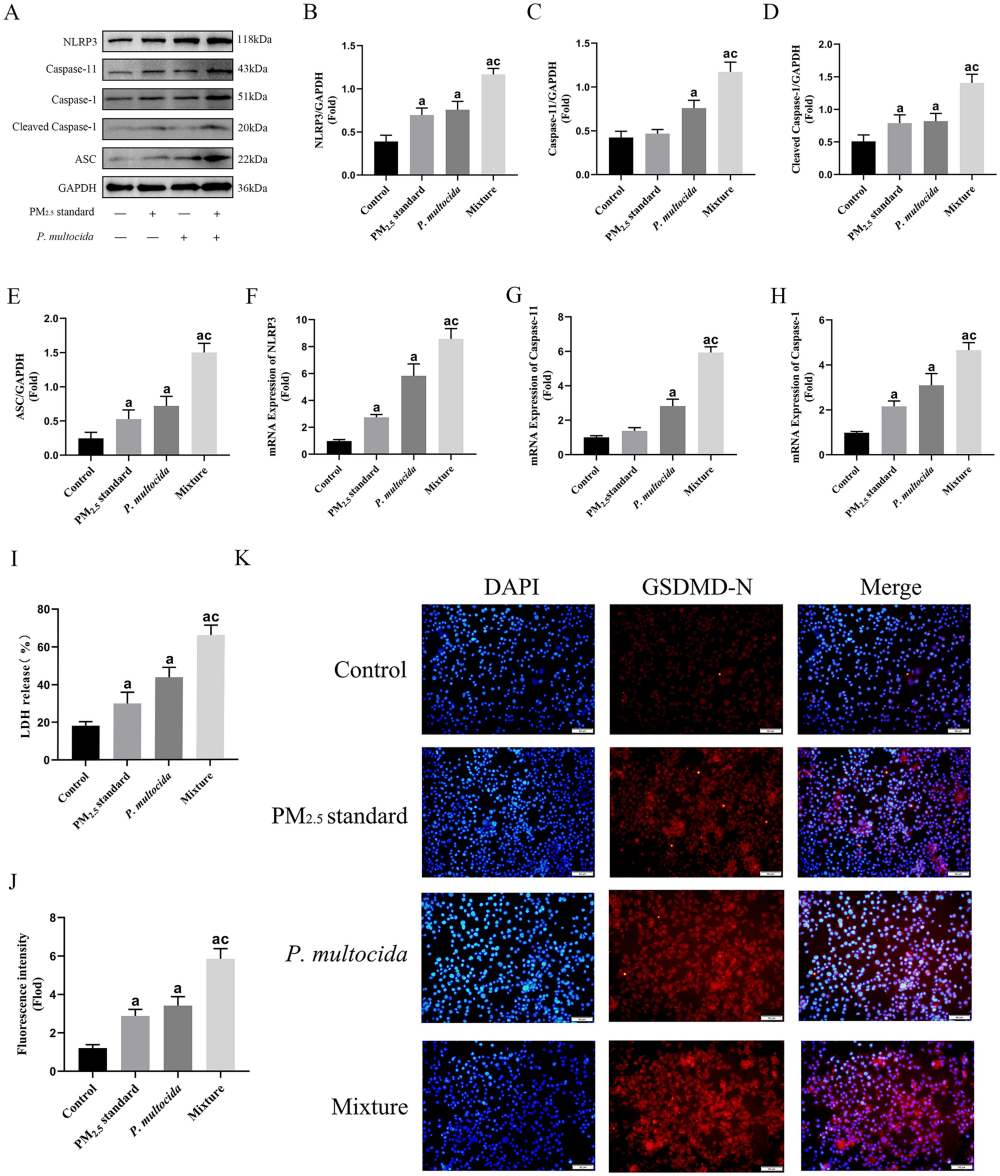
PM_2.5_ enhanced intracellular invasion of *P. multocida* and efficiently initiated Caspase-11-mediated non-classical NLRP3 activation and exacerbated pyroptosis. **(A–E)** Protein expression levels of NLRP3, Caspase-1, Caspase-11, and ASC under the synergistic effect of PM_2.5_ standard and *P. multocida*. **(F–H)** The mRNA expression levels of NLRP3, Caspase-1, Caspase-11 under the synergistic effect of PM_2.5_ standard and *P. multocida*. **(I)** LDH levels under the synergistic effect of PM_2.5_ standard and *P. multocida*. **(J,K)** GSDMD levels under the synergistic effect of PM_2.5_ standard and *P. multocida*. Results are expressed as mean ± SD deviation of three determinations. ^a^*p* < 0.05, compared to the Control group. ^c^*p* < 0.05, compared to the PM_2.5_ standard group.

In order to further validation of the above results, siRNA was used to silence GBP2 expression. First, the silencing effect was tested for three siRNAs. The results showed that all three siRNAs effectively inhibited GBP2 mRNA expression; however, as siRNA-447 had the best silencing effect (Figures 4F,G). Therefore, siRNA-447 was selected for the following studies. Then, the number of intracellular bacteria was assessed after stimulation of normal and GBP2-silenced cells with cowshed PM_2.5_ for 24 h each. It was found that the number of intracellular bacteria was significantly increased in the GBP2-silenced group compared to the unsilenced group (Figure 5H). This suggests that GBP2 plays an important role in the intracellular bacterial defense of host cells against cowshed PM_2.5_ invasion. In addition, during the above process, cell viability of the silenced group was relatively high compared to the unsilenced group for 12 h. However, after 12 h, cell viability plummeted, and after 24 h, cell viability of the unsilenced group was higher than that of the silenced group (Figure 4I). The expression level of IL-1β in the silenced group was initially lower than in the unsilenced group, but over time, expression of IL-1β in the silenced group increased abruptly and was higher than in the unsilenced group (Figure 4J). These data suggest that GBP2 is able to intervene in the cowshed PM_2.5_-induced cytotoxic response, but appears to have complex intrinsic mechanisms.

### 3.5 Recognition of intracellular bacteria by GBP2 activated Caspase-11-mediated non-classical NLRP3

IL-1β is an important effector of NLRP3 expression, and in order to explain the phenomenon of the lag in changes in cell viability and IL-1β expression after GBP2 silencing in the above study. This study focused on investigating the relationship between GBP2 silencing and changes in NLRP3 expression. The results showed that NLRP3 was significantly highly expressed under the effect of cowshed PM_2.5_, whereas silencing GBP2 decreased expression of NLRP3 (Figures 4K–M). This suggests a link between GBP2 and NLRP3. The activation pathway of NLRP3 is relatively complex, and to further explore the relationship between GBP2 and NLRP3, this study focused on the intracellular Caspase-11-mediated non-classical NLRP3 activation pathway. The results showed a relative decrease in the expression of Caspase-11 in the silenced group compared to the unsilenced group (Figures 4N–P), suggesting that GBP2 was associated with the non-classical NLRP3 activation pathway mediated by Caspase-11. Furthermore, silencing or non-silencing of GBP2 had no significant effect on the expression of Caspase-11 and NLRP3 when cells were stimulated with PM_2.5_ standard (Figures 4Q–U). This strongly supports that under the effects of cowshed PM_2.5_, GBP2 intervenes with the hypothesis that Caspase-11-mediated nonclassical NLRP3 activation is associated with the presence of intracellular bacteria. The phenomenon of the lag in changes in cell viability and IL-1β expression after GBP2 silencing may be influenced by the failure to activate Caspase-11 in time to mediate non-classical NLRP3.

### 3.6 PM_2.5_ enhanced intracellular invasion of *Pasteurella multocida*, initiated Caspase-11-mediated non-classical NLRP3 activation, and exacerbates pyroptosis

To further investigate in depth the mechanisms of respiratory toxicity induced by signal transduction in alveolar macrophages in response to intracellular bacterial defenses, similarly, PM_2.5_ standard and *P. multocida* were used to simulate cowshed PM_2.5_. Although only half the dose was used in the Mixture group, in comparison to the PM_2.5_ standard group, expression levels of NLRP3, ASC, cleaved-Caspase-1, and Caspase-11 were elevated (Figures 5A–H). This suggests that during this process, the intracellular transport function of *P. multocida* due to PM_2.5_ particles plays a crucial role in Caspase-11-mediated non-classical NLRP3 activation. This data further elucidates why the Mixture group has an amplifying effect on cytotoxicity in the above results. Activation of the NLRP3 is a significant factor in the induction of pyroptosis, a major manifestation of cellular toxicity, during cellular physiological processes (37). Consequently, alterations in the level of pyroptosis were measured. The results showed that expression levels of GSDMD-N and LDH were significantly increased in the Mixture group compared to the PM_2.5_ standard group (Figures 5I–K). It is shown that pyroptosis is an effective form of cellular damage in the above process and is associated with enhanced intracellular invasion of *P. multocida* by PM_2.5_.

### 3.7 Intervention of NLRP3 expression was an effective respond to cowshed PM_2.5_-induced cellular damage

**Figure 6:**
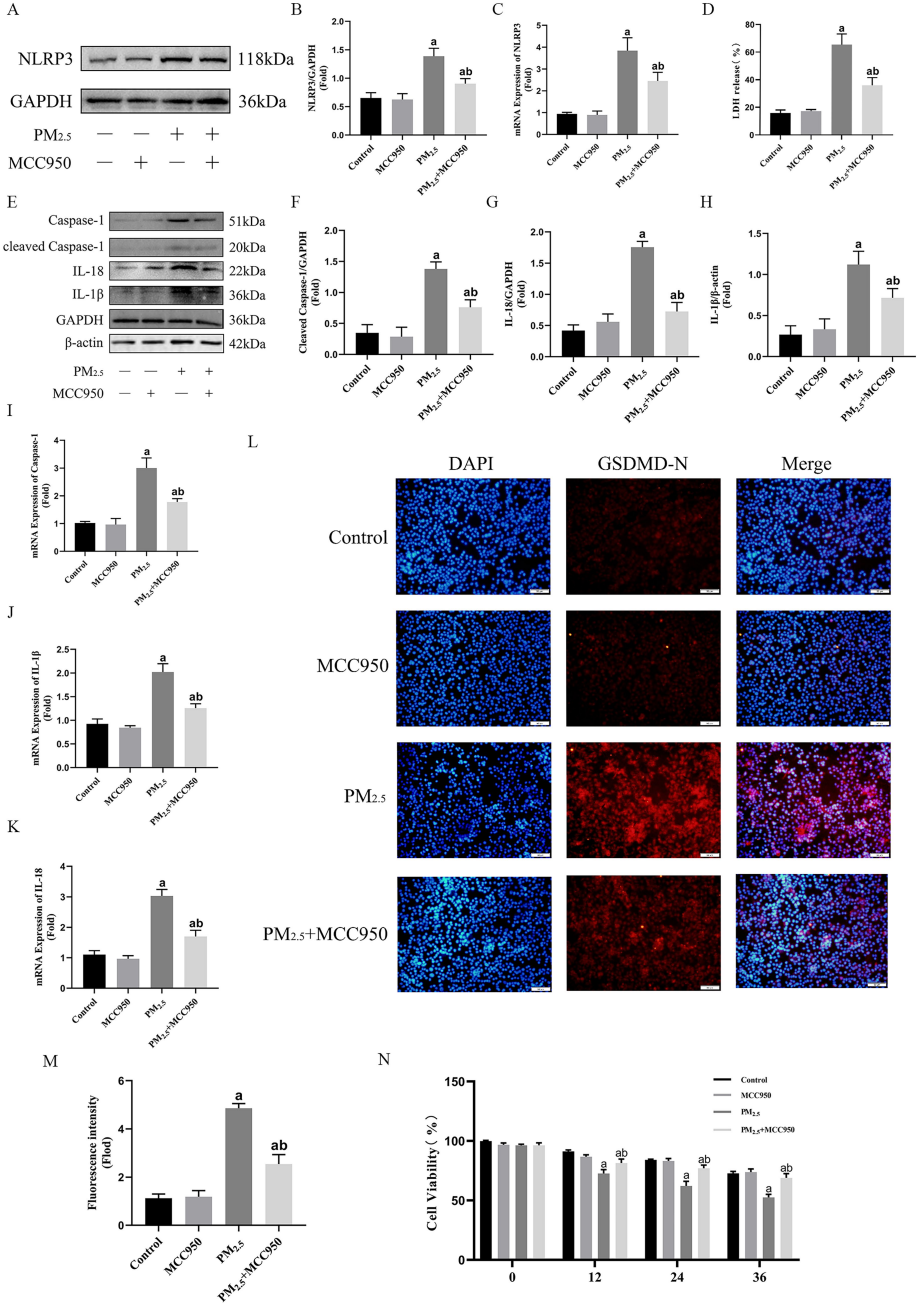
Intervention of NLRP3 expression was an effective respond to cowshed PM_2.5_-induced cellular damage. **(A,B)** Protein levels of NLRP3 after addition of MCC950 under the influence of cowshed PM_2.5_. **(C)** mRNA levels of NLRP3 after addition of MCC950 under the influence of cowshed PM_2.5_. **(D)** LDH levels in NR8383 after addition of MCC950 under the influence cowshed PM_2.5_. **(E–H)** Caspase-1, IL-18, and IL-1β protein expression levels after addition of MCC950 under the influence of cowshed PM_2.5_. **(I–K)** Caspase-1, IL-18, and IL-1β mRNA expression levels after addition of MCC950 under the influence of cowshed PM_2.5._ **(L–M)** GSDMD levels in NR8383 after addition of MCC950 under the influence cowshed PM_2.5_. **(N)** Changes in cell viability levels over time after addition of MCC950 under the influence of cowshed PM_2.5_. Results are expressed as mean ± SD deviation of three determinations. ^a^*p* < 0.05, compared to the Control group. ^b^*p* < 0.05, compared to the PM_2.5_ group.

In this study, although GBP2 silencing initially preserved cell viability, the loss of intracellular bacterial control led to delayed cytotoxicity, indicating that targeting this Gram-negative bacterial defense protein is not a viable therapeutic strategy. Conversely, since cowshed PM_2.5_ triggers Caspase-11-mediated non-classical NLRP3 activation, NLRP3 inhibition was evaluated using an NLRP3 inhibitor (MCC950, MCE). As shown in Figures 6A–C, activation of NLRP3 was significantly inhibited in the PM_2.5_ + MCC950 group compared to the PM_2.5_ group. In addition, the expression levels of cleaved-Caspase-1, IL-1β and IL-18 were also significantly reduced (Figures 6E–K). In terms of pyroptosis, the expression of GSDMD-N and LDH was significantly suppressed in the PM_2.5_ + MCC950 group compared to the cowshed PM_2.5_ group, and cell viability was maintained for an interval in the PM_2.5_ + MCC950 group (Figures 6D,L–N). These data suggest that the use of NLRP3 inhibitors attenuates the onset of pyroptosis and maintains cell viability to some extent, which may be an effective means of treating cowshed PM_2.5_-induced respiratory damage.

## 4 Discussion

### 4.1 Microbial components are critical factors in cowshed PM_2.5_-induced respiratory toxicity

The higher feeding densities and inefficient ventilation in animal farms results in elevates PM_2.5_ concentrations and prolongs exposure longer, thereby increasing the risk of respiratory illness and infection for both animals and workers (38). In the present study, there was substantial pathological damage to the lungs of the model animal (rat) after exposure to cowshed PM_2.5_, accompanied by the release of inflammatory factors. Microorganisms, a major component of PM_2.5_ in the farm-animal environments, are widely present in all aspects of livestock production (39). Furthermore, physiological activities such as ruminating and flatulence are also sources of airborne microorganisms in cowsheds (40); they can utilize particulate matter as a medium for cultivation and transmission. This can result in the emergence of a range of airborne diseases, impairing animal growth, production and welfare, with potential for zoonotic diseases (41). Studies have shown that dairy cows kept for extended intervals in cowsheds with high PM_2.5_ concentrations are more prone to developing respiratory diseases (41). The presence of PM_2.5_ in piggeries has been demonstrated to induce oxidative stress and inflammatory responses in alveolar macrophages, which compromises pig immunity to a certain extent. Furthermore, this can significantly accelerate the course of disease when primary or secondary pathogenic infections are present (42).

Pathogenic bacteria, including *Pseudomonas aeruginosa*, *Shigella escherichii*, *Acinetobacter*, *Streptococcus*, and *Staphylococcus* have been detected in the air of cowsheds. These bacteria pose a serious risk to the organism’s health since they can result in bacteremia or respiratory infections (43). Exposure to PM_2.5_ in the animal farm environment increases the susceptibility of livestock to microorganisms, and this synergistic effect leads to more severe respiratory damage and inflammatory responses (44). In our previous analysis of the composition of cowshed PM_2.5_, bacteria accounted for 61.39% of the microbial composition of cowshed PM_2.5_ (13). Therefore, we speculated that microbiological components had an important role in cowshed PM_2.5_-induced cytotoxicity. In this study, when cytotoxicity experiments were conducted following inactivation of cowshed PM_2.5_, there was a significant decrease in toxicity compared to the not inactivated cowshed PM2.5, confirming the above hypothesis. However, the present study employed high temperatures to inactivate cowshed PM_2.5_, a method that is inherently limited in scope. On the one hand, high temperatures can only transform the microbiologically active components of cowshed PM_2.5_ into inactive components, and do not serve to remove the toxins. On the other hand, circulating high temperatures in localized air within the animal farmhouse is not a widespread practice. Nevertheless, UV light or microwave radiation to address recirculating airflow within the environment has been demonstrated to be an efficacious approach for curbing airborne microbial activity. This may be a viable solution for regulating the biological toxicity of PM_2.5_ and the dissemination of pathogens within the farm-animal environment (45).

### 4.2 Interactions and toxicity amplification mechanisms between PM_2.5_ and bacteria

In addition to this, by analyzing how the microbial component of PM_2.5_ from livestock facilities enhances cytotoxicity, should inform treatment options for animals infected with respiratory diseases. In order to investigate the specific mechanism of microbial components in the induction of cellular damage by cowshed PM_2.5_, we used *P. multocida* isolated from cowshed PM_2.5_ in synergy with PM_2.5_ standard of well-defined compositions to simulate cowshed PM_2.5_. This combination not only excludes as much as possible the unknown effects of the other components, but also shows the characteristics of the cowshed PM_2.5_ as much as possible. Since during PM_2.5_ invasion of the lungs, alveolar macrophages have an important barrier role in the intake and processing of PM_2.5_ particles. Consequently, it is necessary to investigate the response of alveolar macrophages (NR8383) to cowshed PM_2.5_. The experimental results obtained in this study demonstrate that when *P. multocida* is synergized with the PM_2.5_ standard, despite the fact that the stoichiometry is half that of the original, the cellular damage induced by the Mixture group is significantly increased. This also suggests that the microbiological component of PM_2.5_ may play a significant role in the effects on livestock.

In a study of measurements of biomass components in atmospheric PM_2.5_, fungi were found to be the predominant bioactive substances in PM_2.5_, due to the adsorption function of PM_2.5_ particles (46). Therefore, the exploration of the reasons for the strong amplification of PM_2.5_ cytotoxic effects by bacteria may be pivotal in elucidating the mechanism of cowshed PM_2.5_-induced respiratory damage. In order to verify the aforementioned speculations, an examination was conducted of the surface characteristics of particle samples of synergizing PM_2.5_ standard with *P. multocida*, and it was confirmed that *P. multocida* was able to adhere to PM_2.5_ particles. It has been shown that the selenium yeast *A. brasilense* of the genus *Azospirilla* can reduce selenite to basic selenium in the form of selenium nanospheres (47). Silver nanoparticles exhibit significant antimicrobial activity subsequent to interaction with bacterial extracellular polymers (48). This all suggests that there may be a degree of interaction between particulate matter and microorganisms. In this study, the Zeta potential shifted in a negative direction when the PM_2.5_ standard and *P. multocida* were mixed compared to the PM_2.5_ standard group. It has been demonstrated that negatively charged groups secreted by bacteria adsorbed onto the calcite surface induce a shift in the Zeta potential towards a negative direction (49). In the case of *P. multocida*, it has certain surface characteristics and charge distribution itself. After interaction with PM_2.5_, due to adsorption, binding, or other physicochemical processes, a change in the surface charge of PM_2.5_. The shift of the Zeta potential in a negative direction resulted in a reduction in bacterial aggregation and sedimentation, while concurrently enhancing the stability of the PM_2.5_ standard and the *P. multocida* system (50). In the cowshed environment, this would serve to further enhance the spread, survival and pathogenicity of *P. multocida*. Moreover, it would facilitate the more easily formation of bioaerosols, thereby enhancing the toxic effects of PM_2.5_. In the FTIR spectrum, compared to the PM_2.5_ standard group, the red shift of the absorption peak at 3,385 cm^−1^ indicates that the O–H and N–H bond lengths have risen and the bond energy has reduced in the synergistic effect of PM_2.5_ standard and *P. multocida*. This suggests that the two form a stable complex through hydrogen bonding, binding PM_2.5_ to the bacteria. This lays a physical foundation for the subsequent enhancement of intracellular invasion efficiency. Enhancement of the absorption peak at 1,650 cm^−1^ indicates an increase in C=O groups of bacterial origin within the mixture. This is direct evidence of bacterial components being adsorbed onto PM_2.5_ surfaces, thereby altering their chemical composition. This alteration may permit PM_2.5_ to carry more bacterial antigens or toxins (e.g., endotoxin LPS), which in turn enhances immune stimulation. The appearance of the 527 cm^−1^ absorption peak provides further evidence to support that specific chemical binding of bacteria to PM_2.5_ results in the introduction of new toxic groups (e.g., organic acids, cellular metabolites such as carboxyl and hydroxyl groups in proteins) and it changes the PM_2.5_ surface charge distribution, which enhances the retention time of the complex in the respiratory tract and the cell adhesion ability. It has also been shown that in the animal farm environment, PM_2.5_ particles that carry *P. multocida* increase the biological toxicity of the particles themselves. Concurrently, PM_2.5_ particles carrying *P. multocida* are more prone to penetrate the organism’s deeper layers, thereby elevating the probability of disease infection in animals.

### 4.3 Cowshed PM_2.5_ induces cellular pyroptosis and inflammatory responses through activation of the GBP2/Caspase-11/NLRP3 pathway

The present study investigated the effect on the level of intracellular infection of *P. multocida* in the presence of PM_2.5_ particles. It was found that PM_2.5_ greatly increased the probability of intracellular entry of *P. multocida*, which became a pivotal factor in amplifying PM_2.5_ cytotoxicity. However, after PM_2.5_ carries *P. multocida* into cells, the specific mechanism of causing toxicity remains to be elucidated. In this context, bioinformatics and transcriptomics screening revealed a significant up-regulation of GBP2 expression in the lungs in response to cowshed PM_2.5_. GBP2 plays an important role in the immune response. Studies have shown that GBP2 is capable of recognizing and binding to pathogen-associated molecular patterns, thereby initiating immune defense mechanisms (51). Therefore, we speculate that the complex microbiological composition of cowshed PM_2.5_ may be a significant contributing factor to the elevated GBP2 expression. An increase in the number of intracellular bacteria, an enhanced effect of cowshed PM_2.5_ on cell viability, and elevated expression of IL-1β were detected for a period of time following interference with GBP2 expression. This further suggests that microbial components in PM_2.5_ induce high GBP2 expression, and suggests that GBP2 is associated with inflammatory responses induced by microbial components. However, after interfering with GBP2 expression, there was a lag in both cell viability and inflammatory factor expression under the influence of cowshed PM_2.5_, which may be related to the failure of GBP2 to activate Caspase-11 in a timely manner. When GBP2 is silenced, *P. multocida* enters the cell. The clearance of the bacteria by the cell is diminished, and the bacteria continue to multiply intracellularly. However, they have not yet reached the threshold for triggering intense inflammation. This results in a transient increase in cell viability in the initial period (within 12 h). In the present study, it was also detected that after interfering with GBP2, there was no activation of Caspase-11 expression in the presence of cowshed PM_2.5_. However, it has been demonstrated that persistent intracellular bacterial reproduction can ultimately trigger the lagging activation of the non-classical NLRP3 pathway, leading to a surge of inflammatory factors and, ultimately, triggering a sudden drop in cell viability. This phenomenon reveals the pivotal function of GBP2 in maintaining a balance between antimicrobial defense and inflammation control. Furthermore, these findings suggest that the silencing of GBP2, although protective in the short term, leads to more severe damage over time.

Many studies have shown that PM_2.5_ can induce a variety of programmed cell deaths, including apoptosis, autophagy and pyroptosis, and so on (52). However, the relationship and signaling mechanisms between PM_2.5_ and various forms of programmed cell death are complex. Among the various forms of programmed cell death described above, pyroptosis is capable of triggering a more intense inflammatory response, a process that helps to protect the host from microbial infections (53). However, the consequences of excessive pyroptosis are also evident, with excessive cellular pyroptosis leading to a series of inflammatory storms that can cause sepsis and autoimmune diseases (54). Therefore, it is crucial to explore the mechanism of pyroptosis induced by cowshed PM_2.5_. Studies have shown that upon bacterial entry into cells, GBP2 cleaves pathogen-containing vesicles (PVs), thereby releasing bacteria and their associated LPS into the host cell cytoplasm and assembling a Caspase-11 activation platform on LPS-containing membranes as the first step of the inflammasome signaling, and activating the non-classical NLRP3 inflammasome (55). The mechanism of activation of the NLRP3 inflammasome is similarly intricate. Studies have shown that chemicals endocytosed into the cytoplasm by macrophages trigger lysosomal rupture and the release of histone B, leading to activation of the NLRP3 inflammasome (56). The rupture of the cytoplasmic membrane with K^+^ efflux caused by the PAHs component contained in PM_2.5_ may also be a significant reason for the activation of NLRP3 (57). In this study, the expression of NLRP3 and Caspase-11 did not change significantly after interfering with the expression of GBP2 under the role of PM_2.5_ standard. However, interference with GBP2 was found to have a substantial impact on the expression of NLRP3, cleaved-Caspase-1, Caspase-11, GSDMD, and LDH, under the synergistic effect of PM_2.5_ standard and *P. multocida*. These results provide validation for the upregulation of GBP2 expression upon the entry of cowshed PM_2.5_ into cells. Subsequently, GBP2 is able to recognize and bind to microbial components in cowshed PM_2.5_, further affecting the activation of the NLRP3 non-classical pathway mediated by Caspase-11.

### 4.4 Inhibition of NLRP3 can stably suppresses cowshed PM_2.5_-induced respiratory toxicity

In this study, it was determined that GBP2 is not an effective target against cowshed PM_2.5_-induced pyroptosis. Therefore, we focused our perspective on NLRP3, the activation of which is the central channel for pyroptosis caused by cowshed PM_2.5_ in the present study. In this regard, the present study attempted a therapeutic option to reduce cowshed PM_2.5_-induced damage by inhibiting NLRP3. The development of cellular inflammation and pyroptosis was effectively controlled by using an NLRP3 inhibitor (MCC950) to inhibit NLRP3 expression. This demonstrates the effectiveness of this regimen. This also provides a valuable therapeutic target and dosing direction for the treatment of cowshed PM_2.5_-induced lung injury. However, there are some limitations to this study. This study only assessed the interrelationships and mechanisms of action between PM_2.5_ and bacteria, and therefore does not reflect the full picture of the microbiological landscape within cowshed PM_2.5_. This encompasses the potential involvement of disease-causing microorganisms (such as fungi, viruses, and parasites) in the process of damage to organisms. Further research is required to elucidate this aspect, which will be the focus of our subsequent studies.

## 5 Conclusion

In summary, the present study demonstrated that microbiological components were non-negligible and important factors in animal farm environment PM_2.5_-induced lung injury. Bacterial components alter the surface characteristics of PM_2.5_ particles, whereas PM_2.5_ particles enabled bacteria to get inside the cells. The interaction between the two amplified the biological toxicity of PM_2.5_. Intracellularly, recognition and defense of bacteria by GBP2 activated pyroptosis induced by non-classical NLRP3. The present study elucidated the relationship among intracellular bacteria, GBP2, and NLRP3 as a key process in cowshed PM_2.5_-induced pyroptosis. In addition, inhibition of NLRP3 has potential for the treatment of PM_2.5_-induced lung injury in a farm-animal environment.

## Data Availability

The original contributions presented in the study are included in the article/Supplementary material, further inquiries can be directed to the corresponding authors.
