# Supplementary material for: Interactions between particulate matter and bacteria during cowshed PM2.5-induced respiratory injury initiates GBP2/Caspase-11/NLRP3-mediated intracellular bacterial defense and pyroptosis
Source: Front Vet Sci. 2025 Jul 8;12:1631913. doi: 10.3389/fvets.2025.1631913 (PMC12280994; doi:10.3389/fvets.2025.1631913)
Supplement: Supplementary file 1 [file Table_1.docx]

| **component** | **Content（%）** |
| --- | --- |
| O | 56.7 |
| Na | 6.69 |
| Mg | 1.47 |
| Al | 3.6 |
| Si | 10.5 |
| P | 0.37 |
| S | 5.91 |
| Cl | 3.65 |
| K | 1.09 |
| Ca | 6.81 |
| Ti | 0.30 |
| Fe | 2.52 |
| Cu | 0.46 |

**Supplementary Table S1**

**Table S1. The composition of the PM2.5 standard**

**Supplementary Table S2**

| **Instruments and reagents** | **Source** | **Batch** |
| --- | --- | --- |
| Multi-level flow particulate sampler | Laoying Haina Photovoltaic Environmental Protection Group Co., Qingdao | 2030 |
| PM_2.5_ standard | JRC Science Hub | ERM-CZ110 |
| Microscope | Phenix, China |  |
| 10% fetal bovine serum | Beyotime Biotechnology, Shanghai, China | C0235 |
| 1% penicillin-streptomycin | Beyotime Biotechnology, Shanghai, China | C0222 |
| MCC950 | MCE, China | HY-12815A |
| CCK-8 kit | Beyotime Biotechnology, Shanghai, China | C0037 |
| Enzyme labeler | Thermo Fisher, USA |  |
| ROS | Nanjing Jiancheng Bioengineering Institute | E004-1-1 |
| MDA | Nanjing Jiancheng Bioengineering Institute | A003-4-1 |
| SOD | Nanjing Jiancheng Bioengineering Institute | A001-1-1 |
| Lipofectamine 3000 | Thermo Fisher, MA, USA | L3000015 |
| *ELISA* kit | Jiangsu Meimian Industrial Co., Ltd., China | ml037361 |
| LDH cytotoxicity assay kit | Beyotime, Shanghai, China | C0016 |
| GSDMD-N antibody | abcam, USA | 1:100, ab215203 |
| CY3-coupled secondary antibodies | Proteintech | 1:100, SA00009-2 |
| Fluorescence microscope | Olympus |  |
| The Total RNA Extraction Kit | Sangon Biotech, China | B511311 |
| The PrimeScript™ RT Kit | TaKaRa, Kyoto, Japan | RR047A |
| RIPA lysate | Sangon Biotech, Shanghai, China | C500005 |
| BCA protein quantification kit | Beyotime Biotechnology, China | P0010S |
| PVDF membranes | Millipore, USA | GVWP04700 |
| ECL luminescence assay kit | Monad, USA | PW30601S |
| Amersham Imager 680 | GE Healthcare, Sunnyvale, Chicago, Illinois, USA |  |
| NLRP3 antibody | ABclonal, China | A24294 |
| GAPDH antibody | ABclonal, China | AC001 |
| Caspase-1 antibody | Abcam, USA | ab138483 |
| cleaved Caspase-1 antibody | Cell Signaling Technology | #4199 |
| Caspase-11 antibody | Affinity Biosciences, China | DF7609 |
| GBP2 antibody | Proteintech Group, China | 11854-1-AP |
| ASC antibody | Proteintech Group, China | 10500-1-AP |
| IL-18 antibody | Proteintech Group, China | 10663-1-AP |
| IL-1β antibody | Proteintech Group, China | 16806-1-AP |
| β-actin antibody | Proteintech Group, China | 20536-1-AP |
| Secondary antibodies (Goat Anti-Rabbit IgG) | Proteintech Group, China | SA00001-2 |

Note: Consistent with the order in materials and methods in the study.

**Table S2. Detailed information on Instruments and reagents in the article**
